# Combination of a Chaperone Synthesis Inducer and an Inhibitor of GAPDH Aggregation for Rehabilitation after Traumatic Brain Injury: A Pilot Study

**DOI:** 10.3390/pharmaceutics15010007

**Published:** 2022-12-20

**Authors:** Elizaveta A. Dutysheva, Elena R. Mikhaylova, Maria A. Trestsova, Alexander I. Andreev, Danila Yu. Apushkin, Irina A. Utepova, Polina O. Serebrennikova, Eugenia A. Akhremenko, Nikolay D. Aksenov, Elizaveta I. Bon’, Sergey M. Zimatkin, Oleg N. Chupakhin, Boris A. Margulis, Irina V. Guzhova, Vladimir F. Lazarev

**Affiliations:** 1 Institute of Cytology of the Russian Academy of Sciences, 194064 St. Petersburg, Russia; 2 Department of Organic and Biomolecular Chemistry, Ural Federal University, 620002 Ekaterinburg, Russia; 3 Laboratory of Experimental Pharmacology, Perm State University, 614990 Perm, Russia; 4 Perm State Pharmaceutical Academy, 614990 Perm, Russia; 5 Postovsky Institute of Organic Synthesis, Ural Branch, The Russian Academy of Sciences, 620108 Ekaterinburg, Russia; 6 Department of Histology, Cytology and Embryology, Grodno State Medical University, 230009 Grodno, Belarus

**Keywords:** traumatic brain injury, heat shock protein, Hsp70, GAPDH, small molecules, secondary damage, rehabilitation therapy, hydrocortisone 21-hemisuccinate, 3-(5-phenyl-1H-pyrrol-2-yl)quinoxalin-2(1H)-one

## Abstract

The recovery period after traumatic brain injury (TBI) is often complicated by secondary damage that may last for days or even months after trauma. Two proteins, Hsp70 and glyceraldehyde-3-phosphate dehydrogenase (GAPDH), were recently described as modulating post-traumatic processes, and in this study, we test them as targets for combination therapy using an inhibitor of GAPDH aggregation (derivative of hydrocortisone RX624) and an inducer of Hsp70 synthesis (the pyrrolylazine derivative PQ-29). The protective effect of the combination on C6 rat glioblastoma cells treated with the cerebrospinal fluid of traumatized animals resulted in an increase in the cell index and in a reduced level of apoptosis. Using a rat weight drop model of TBI, we found that the combined use of both drugs prevented memory impairment and motor deficits, as well as a reduction of neurons and accumulation of GAPDH aggregates in brain tissue. In conclusion, we developed and tested a new approach to the treatment of TBI based on influencing distinct molecular mechanisms in brain cells.

## 1. Introduction

**Figure 1:**
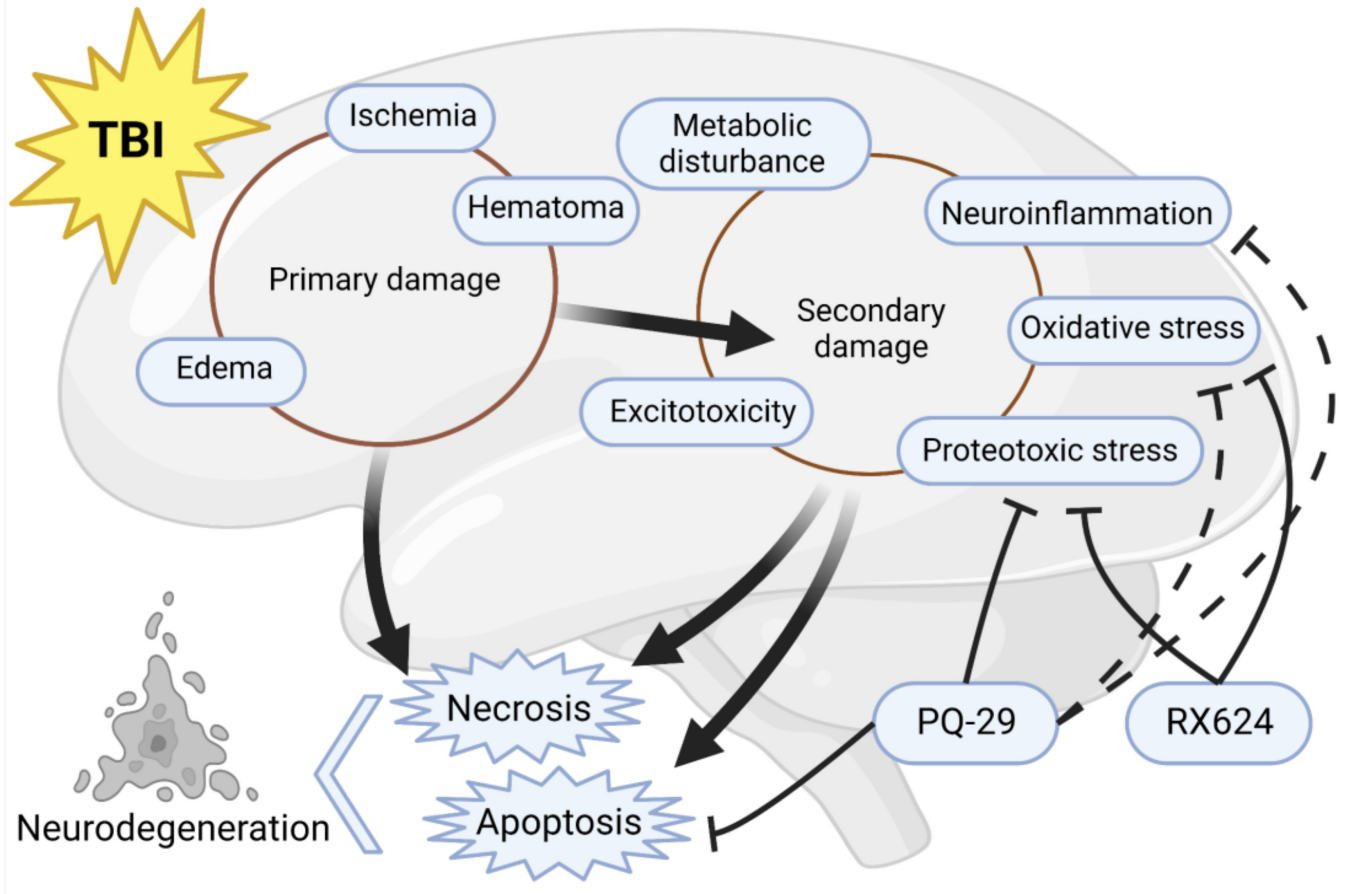
Influence of drugs RX624 and PQ-29 on pathological mechanisms developing after TBI. Continuous lines show the previously proven effect of PQ-29 and RX624; dotted lines show the probable (unexplored) effect of PQ-29. Created with BioRender.com.

Traumatic brain injury (TBI) is one of the leading causes of death and disability among people regardless of age [1]. The consequences of TBI vary considerably from complete recovery to severe coma and the death of the victim. Mortality in patients with severe TBI reaches 40%, with the remaining 60% experiencing a significant decrease in their quality of life, especially in the context of significant socio-economic costs [2]. The consequences of TBI at the cellular level include inflammation; a burst of reactive oxygen species; protein aggregation (proteotoxic stress); and finally, massive cell death regulated by apoptosis mechanisms [3,4] (Figure 1).

TBI is a factor that causes attributes reminiscent of Alzheimer’s disease (AD), chronic traumatic encephalopathy [5], Parkinson’s disease [6], amyotrophic lateral sclerosis [7], and others. This feature makes combating the pathogenesis of TBI much more relevant.

Significantly, TBI-induced cell death does not always follow immediately after trauma; for example, cell apoptosis is detected 48–72 h after injury [8]. Delayed cell death is not a predestined and irreversible process, leaving ample time for therapeutic intervention. The development of the pathogenesis of TBI is difficult to predict, and therefore, the use of known drugs is not always effective. A number of authors believe that the key to success is the use of combined therapy aimed at suppressing parallelly developing pathogenic processes [9].

One of the components of cell protection in neurodegenerative and inflammatory processes is the chaperone system, including the Hsp70 protein. Hsp70 prevents the development of caspase-dependent [10] and/or -independent pathways of apoptosis [11], is known to recognize degradation proteins with an abnormal conformation, and is also subjected to degradation [12]. Hsp70 is also known to mediate the cell response to pro-inflammatory cytokines via TLRs and scavenger receptors [13]. The major property of Hsp70 is its chaperonic activity, i.e., its ability to bind denatured or misfolded proteins and to prevent their oligomerization and aggregation [14]. Thus, Hsp70 can affect the pathogenic processes caused by TBI through multiple interactions with cellular proteins; the result of such reactions is an increased survival of brain cells and a restoration of neuronal function. We have previously shown that inducers of heat shock protein synthesis, including Hsp70, can prevent neurodegeneration in in vitro and in vivo [15] TBI models. One of these potential therapeutic agents may be a PQ-29 compound belonging to the class of pyrrolylazines, for which we have demonstrated a therapeutic effect in Alzheimer’s disease and TBI [16] models (Figure 1).

Another target for therapeutic intervention is glyceraldehyde-3-phosphate dehydrogenase (GAPDH), which can form cytotoxic aggregates in cells subjected to oxidative stress [17]. One of these proteins, glyceraldehyde-3-phosphate dehydrogenase (GAPDH), can form cytotoxic aggregates as a result of oxidative stress [18]. The enzyme can also form co-aggregates with known pathogenic proteins, such as beta-amyloid [19], mutant huntingtin [20], and mutant superoxide dismutase [21]. We have previously established that TBI leads to the formation of GAPDH aggregates in brain tissues and in the CSF of animals. Therefore, it appears that small molecules capable of binding GAPDH may prevent its participation in the formation of such toxic aggregates. In particular, the hydrocortisone derivative RX624 [22,23], found to block the formation of GAPDH complexes with other protein pathogens in the intercellular space, has a similar neuroprotective potential (Figure 1).

The data on the two presented targets show the promise of an approach that combines an increase in the anti-apoptotic and anti-inflammatory activities of Hsp70 with the suppression of the formation of GAPDH-containing aggregates (Figure 1). The investigation of this new therapeutic approach is the aim of the present study. Using our previously developed models of TBI in vitro and in vivo, we evaluated the effectiveness of this combination therapy on the effects of TBI.

## 2. Materials and Methods

### 2.1. Cells

Rat glioblastoma C6 cells were obtained from the shared research facility “Vertebrate cell culture collection” supported by the Ministry of Science and Higher Education of the Russian Federation (Agreement No. 075-15-2021-683). Cells were cultured in DMEM/F12 medium (Gibco, Carlsbad, CA, USA) containing 10% fetal bovine serum (FBS; Gibco, Carlsbad, CA, USA), 100 units/mL penicillin, and 0.1 mg/mL streptomycin (BioloT, St. Petersburg, Russia) at 37 °C and 5% CO_2_.

### 2.2. Chemical Compounds

Hydrocortisone 21-hemisuccinate compound (RX624) was purchased from the European Directorate for the Quality of Medicines & HealthCare (Strasbourg, France).

The compound 3-(5-phenyl-1H-pyrrol-2-yl)quinoxalin-2(1H)-one (PQ-29) was synthesized by us, according to the previously described procedure [24].

**Scheme 1:**
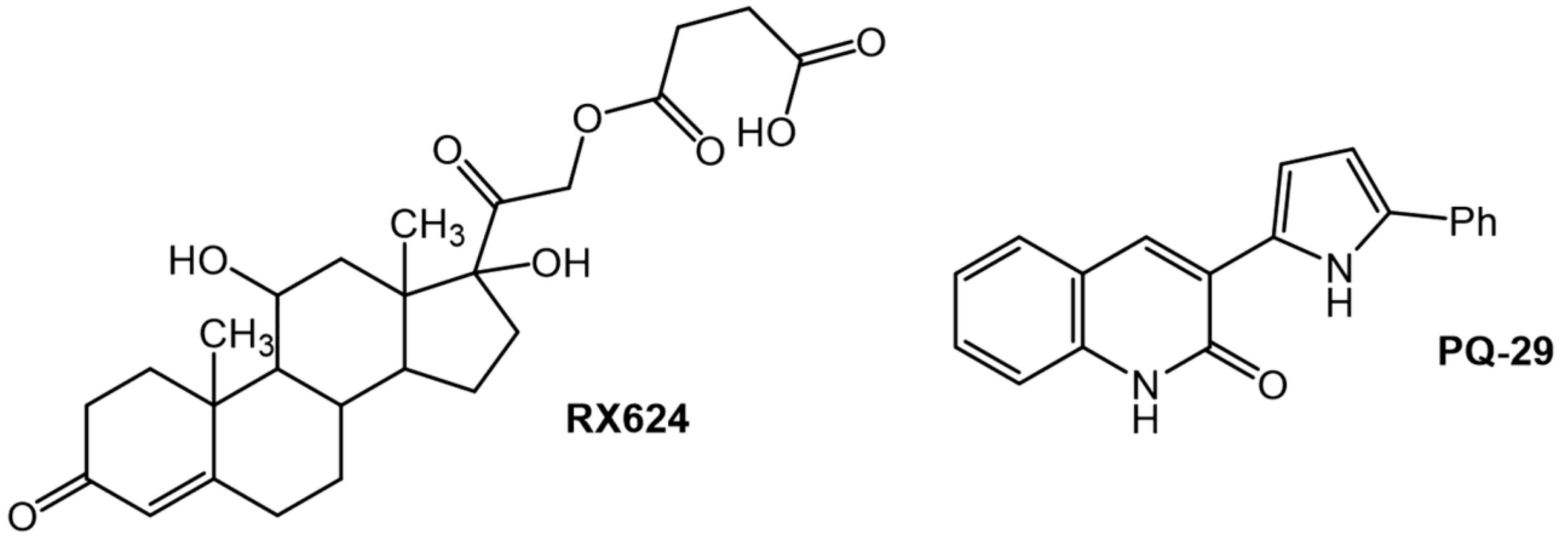
Chemical structure of hydrocortisone 21-hemisuccinate compound (RX624) and 3-(5-phenyl-1H-pyrrol-2-yl)quinoxalin-2(1H)-one (PQ-29).

DMSO was used as a vehicle for both compounds. The chemical structures of the compounds presented with the ChemSketch 2.1 software (ACD/Labs, Toronto, Canada) are shown in Scheme 1.

### 2.3. Modeling of Traumatic Brain Injury

Male Wistar rats from the Rappolovo livestock breeding complex (Russia) weighing 200–250 g on the 75th–80th days of life were used in this study. TBI was induced with the aid of a weight drop (150 g) according to the protocol of Mychasiuk et al. [25] with a slight difference: the fall height was 120 cm (instead of 100 cm) [15]. For the cell experiments, CSF was collected through the foramen magnum 3 days after injury. CSF from untraumatized animals was used as a control. To analyze the therapeutic potential of the combined use of PQ-29 and RX624 in animals, Wistar rats weighing 200–250 g were divided into five groups: untraumatized animals (control, n = 6); traumatized animals (TBI, n = 6); traumatized and treated with PQ-29 (PQ-29, n = 6); traumatized and treated with RX624 (RX524, n = 6); and traumatized and treated with a combination of PQ-29 and RX624 (PQ-29 + RX624, n = 6). Therapy was performed using intraperitoneal injections of PQ-29 at a dosage of 1 mg/kg and/or RX624 at a dosage of 1 mg/kg 3 times a week for 30 days.

All animal experiments were conducted in agreement with the Ethics Committee of the Institute of Cytology RAS No. F18-00380 (approved on 12 October 2017).

### 2.4. Cell Proliferation Assay

Real-time assessment of C6 cell proliferation was performed using the xCELLigence RTCA DP instrument (ACEA Biosciences, Inc., San Diego, CA, USA). To analyze the cell index, C6 cells (10^5^ cells per mL) were added to the wells of a 16-well E-board, at the bottom of which a gold electrode was placed. C6 cells were incubated in a growth medium mixed with the CSF of traumatized animals at a 1:1 volume ratio, and a cell index evaluation (measurement of cell resistance) was performed every 15 min, as we described earlier [15]. Recording was conducted for 3 days. The results were analyzed using the xCELLigence RTCA DP instrument software, Software 1.2. (ACEA Biosciences, Inc., San Diego, CA, USA).

### 2.5. Apoptotic Cell Analysis

The percentage of apoptotic cells was determined using the Annexin V TM 647 kit (Life Technologies, Eugene, OR, USA). C6 cells were incubated in a growth medium mixed with the CSF of traumatized animals at a 1:1 volume ratio for 6 h. The cells were harvested, washed with phosphate buffer, and then stained with annexin V Alexa Fluor 647 and propidium iodide according to the manufacturer’s protocol. The cell suspension was analyzed by flow cytometry on a CytoFlex flow cytometer (Beckman Coulter, Miami, FL, USA). For detection, lasers with wavelengths of 488 (propidium iodide fluorescence) and 638 nm (Alexa 647 fluorescence) were used. The data obtained were analyzed using the CytExpert 2.0 software (Beckman Coulter, Miami, FL, USA).

### 2.6. Morris Water Test

Memory impairment in rats was assessed using the Morris water test [26] with a diameter of 1.5 m (OpenScience, Krasnogorsk, Russia) on the 30th day after TBI. The test consisted of two parts: training and testing. For 5 consecutive days, the rats were trained to find the platform. Daily training included 4 attempts with an interval of 15 s. On the 6th day, their spatial memory was tested. To achieve this, the platform was removed, the animal was placed in the sector that was the most distant from the target, and its movements were traced for one minute. Tracing was conducted using the EthoStudio program, which calculated the following indicators: (1) the path (cm) that the animal used for 60 s and (2) the time spent by the animal in each of the sectors. Then, the average value of the animal’s stays in non-target sectors was calculated and compared to the time spent in the target sector.

### 2.7. Analysis of the Locomotor Function of Rats

Movement disorders were assessed using the beam-walking test (OpenScience, Russia) on the 37th day after TBI. The testing procedure was recorded on video. Motor function was assessed by determining the percentage of incorrect paw placements, and slips and falls of the animals from the track, which were calculated as previously described [22,27].

### 2.8. Immunohistochemistry

At the end of the beam-walking test (41 days after TBI), the rats were anesthetized with Zoletil-100 (50 mg/kg, intraperitoneal), perfused with 4% paraformaldehyde, and then decapitated. The brain was extracted and examined by confocal microscopy. Brains from all animals used for immunohistochemical assays were fixed in 4% paraformaldehyde and cryoprotected in 20% sucrose before storage in isopentane at −70 °C. Coronal sections (15 μm) were prepared for morphological and immunohistochemical assay with a Leica CM1510S-1 cryostat (Leica Microsystems, Wetzlar, Germany). The frontal slices were collected at the level of the hippocampus (from bregma −3 mm to −4.3 mm) according to the atlas of Paxinos and Watson [28]. Six alternate series of sections were mounted on SuperFrost Plus slides (Menzel GmbH, Berlin, Germany). For confocal microscopy, sections were preincubated in blocking solution (2% bovine serum albumin diluted in PBS with 0.1% Tween-20) for 1 h at room temperature and then hybridized with anti-NeuN (Abcam, Cambridge, UK) or anti-GAPDH antibodies (Elabscience, Houston, TX, USA) following hybridization with secondary fluorescently labeled antibodies (Thermo Fisher Scientific, Waltham, MA, USA). After rinsing in PBS, the sections were subsequently incubated in 40,6-diamidino-2-phenylindole (DAPI) for 10 min (1:10,000; Sigma-Aldrich, St. Louis, MO, USA). Fluorescent images were captured by an Olympus FV3000 confocal system (Olympus, Tokyo, Japan). Surviving cells were counted based on DAPI staining using the ImageJ software. At least 500 cells were counted for each point.

### 2.9. Aggregate Detection

To analyze the amount of aggregates containing GAPDH in the rat hippocampus, we used the ultrafiltration method (filter trap assay) previously described in [23]. Rat hippocampal lysates were dissolved in a buffer (10 mM Tris-HCl pH 8.0, 150 mM NaCl, and 2% sodium dodecyl sulfate) in the amount of 200 μg of total protein and applied to a cellulose acetate membrane placed in an ultrafiltration apparatus attached to a vacuum pump (BIO-RAD, Hercules, CA, USA). Before and after applying the lysates, the membrane was washed under pressure with a buffer of the following composition: 10 mM Tris-HCl pH 8.0, 150 mM NaCl, and 0.1% sodium dodecyl sulfate. The presence of GAPDH in the aggregates was determined using specific antibodies, clone 6C5 in a concentration of 0.5 μg/mL (Abcam, Cambridge, UK), followed by hybridization with secondary antibodies labeled with peroxidase (1:10,000; Jackson Laboratory, Farmington, CT, USA). Using the TotalLabQuant software, we obtained the dot intensity value in conventional units, which was then normalized to the mean GAPDH staining intensity in the hippocampus of naive rats.

### 2.10. Statistical Analysis

All data were expressed as mean ± standard deviation. Data were compared using a non-parametric Mann–Whitney test using the GraphPad Prism 8 software. All experiments, except animal studies, were repeated at least three times. Statistical difference was determined by *p* < 0.05.

## 3. Results

### 3.1. Effect of the Combined Use of RX624 and PQ-29 Preparations on the Physiological Parameters of C6 Cells in an In Vitro TBI Model

**Figure 2:**
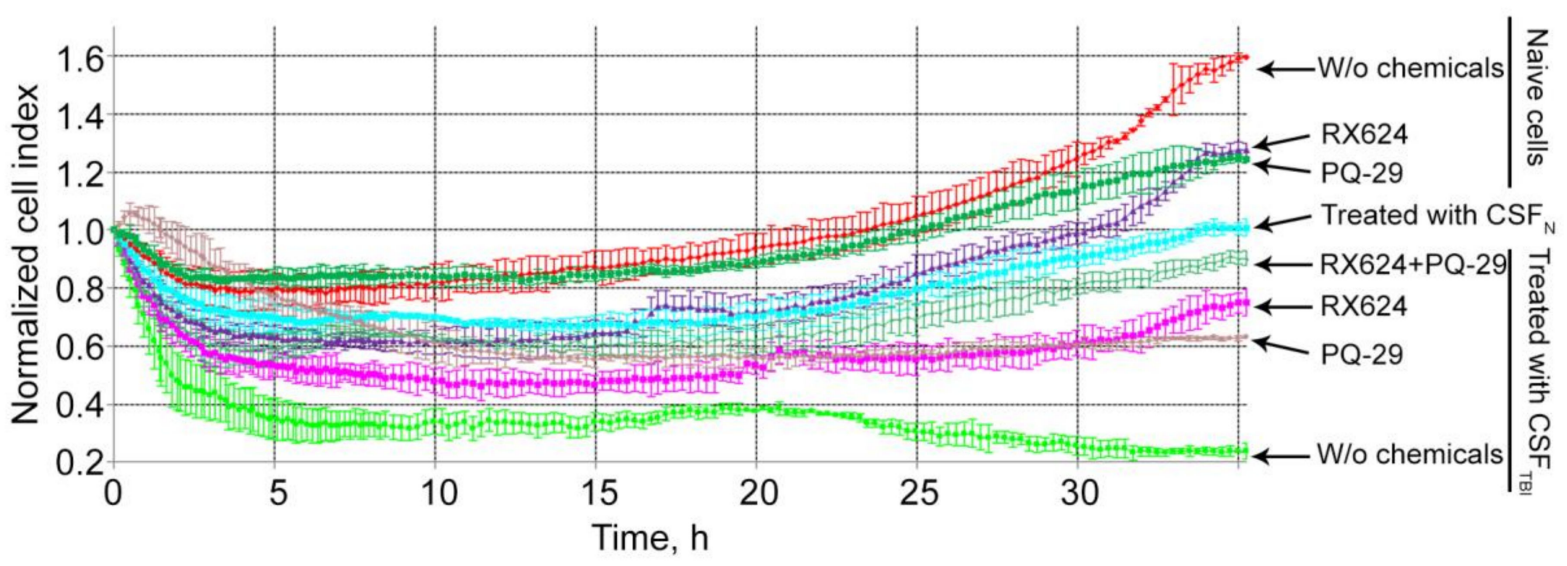
Dynamics of cellular index of C6 rat glioma cells cultivated in the presence of rat CSF, as well as RX624 and PQ-29 compounds. The data obtained using the xCELLigence setup are presented. CSF_N_—CSF obtained from non-traumatized animals; CSF_TBI_—CSF obtained from traumatized animals.

**Figure 3:**
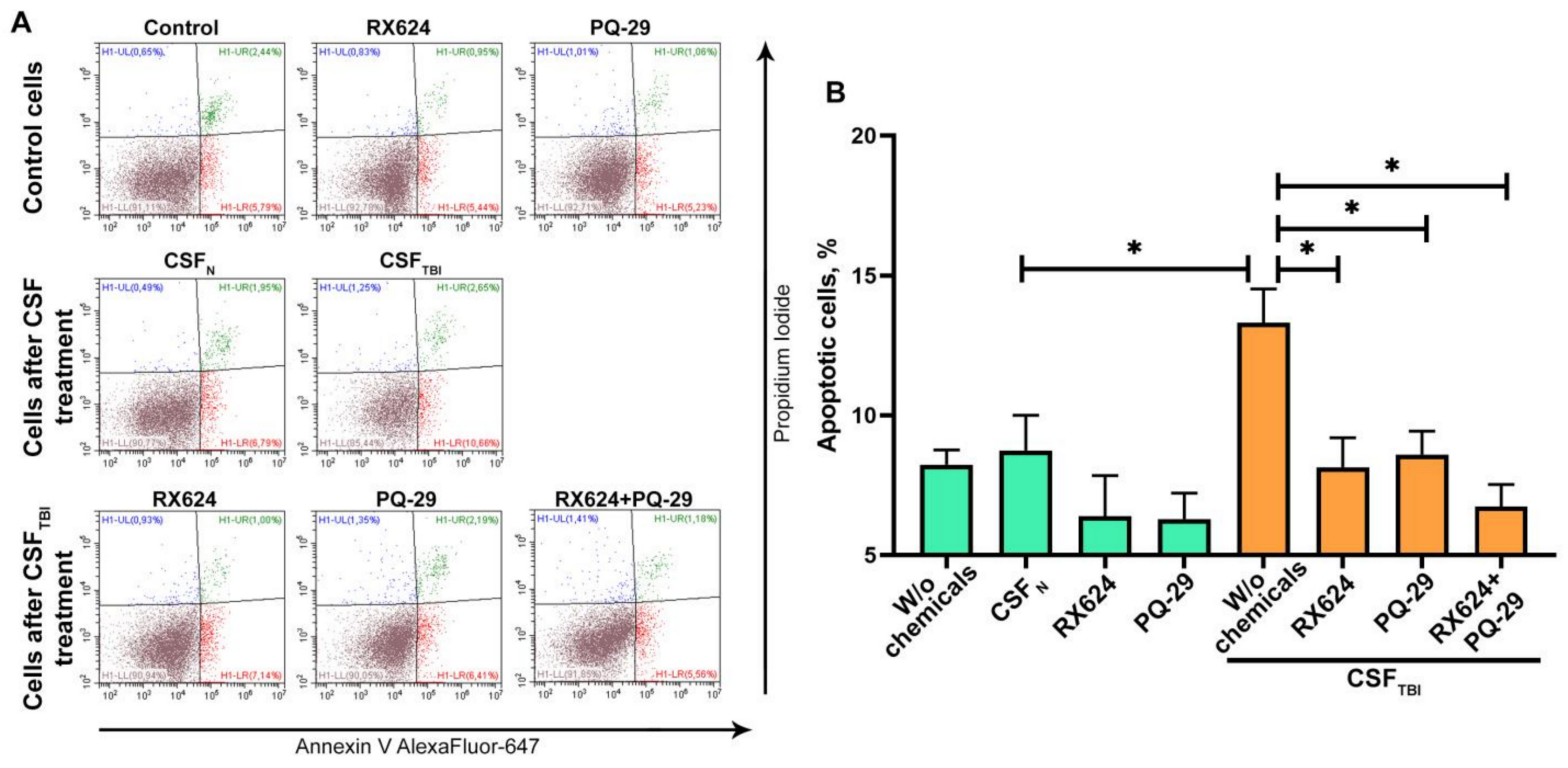
Analysis of the proportion of apoptotic cells in the population after incubation in the presence of CSF of traumatized rats and therapeutic compounds. CSF_N_ is the cerebrospinal fluid of naive animals. CSF_TBI_ is the cerebrospinal fluid of traumatized animals. (**A**) Distribution of the cell population according to flow cytometry data after cell staining with the apoptosis marker annexin V. (**B**) The results of analysis using the flow cytometry method are presented as a bar graph. The mean values ± standard deviations of the results of three independent experiments are shown; differences are significant at * *p* < 0.05 (Mann–Whitney test).

During the first stage of the work, we tested the effectiveness of the therapeutic use of a combination of PQ-29 and RX624 (the chemical structures of the compounds are shown in Scheme 1) drugs on an in vitro TBI model. We used a previously designed TBI-based model, in which C6 rat glioma cells were incubated in the presence of the CSF of traumatized animals; this incubation inhibited cell growth and increased the level of cell death, events that usually accompany the post-trauma period [15]. To estimate the efficacy of combination therapy, we supplemented the CSF of traumatized animals with RX624 and PQ-29. Cell viability was determined using the cell index monitoring system xCELLigence (Figure 2), while the level of apoptosis was estimated by the staining of phosphatidylserine with annexin V (Figure 3).

We discovered that culturing cells with the CSF of traumatized animals in the presence of 1 μM PQ-29 and 1 µM RX624 resulted in a 44% recovery of the cell index compared to C6 cells cultured only with CSF after TBI. At the same time, the separate use of an inhibitor of exogenous GAPDH—RX624—and an inductor of HSP70 synthesis—PQ-29—led to the restoration of the cell index by 17% and 20%, respectively (Figure 2).

Similarly, an analysis of the amount of apoptotic cells using annexin staining showed that, compared to the use of pyrrolylazine and a hydrocortisone derivative separately (8.14% and 8.6%, respectively), their combined use in a model of CSF-mediated damage after TBI led to a reduction in the apoptosis level from 13.31% to 6.74% (Figure 3).

Thus, the combination therapy tended to be more effective in preventing the deterioration of C6 rat glioma cells compared to both versions of monotherapy.

### 3.2. Effect of Combination Therapy Using RX624 and PQ-29 on the Behavioral, Hystological and Physiological Characteristics of Traumatized Rats

Next, we sought to test combination therapy using the RX624 and PQ-29 compounds in a TBI model in rats. The experimental animals were divided into five groups: (1) naive rats that did not receive any treatment; (2) traumatized rats; (3) traumatized rats treated with RX624; (4) traumatized rats treated with PQ-29; and (5) traumatized rats that received combined treatment with RX624 and PQ-29. The duration of the therapeutic course was 30 days. Following this time period, the experimental animals were subjected to behavioral tests.

**Figure 4:**
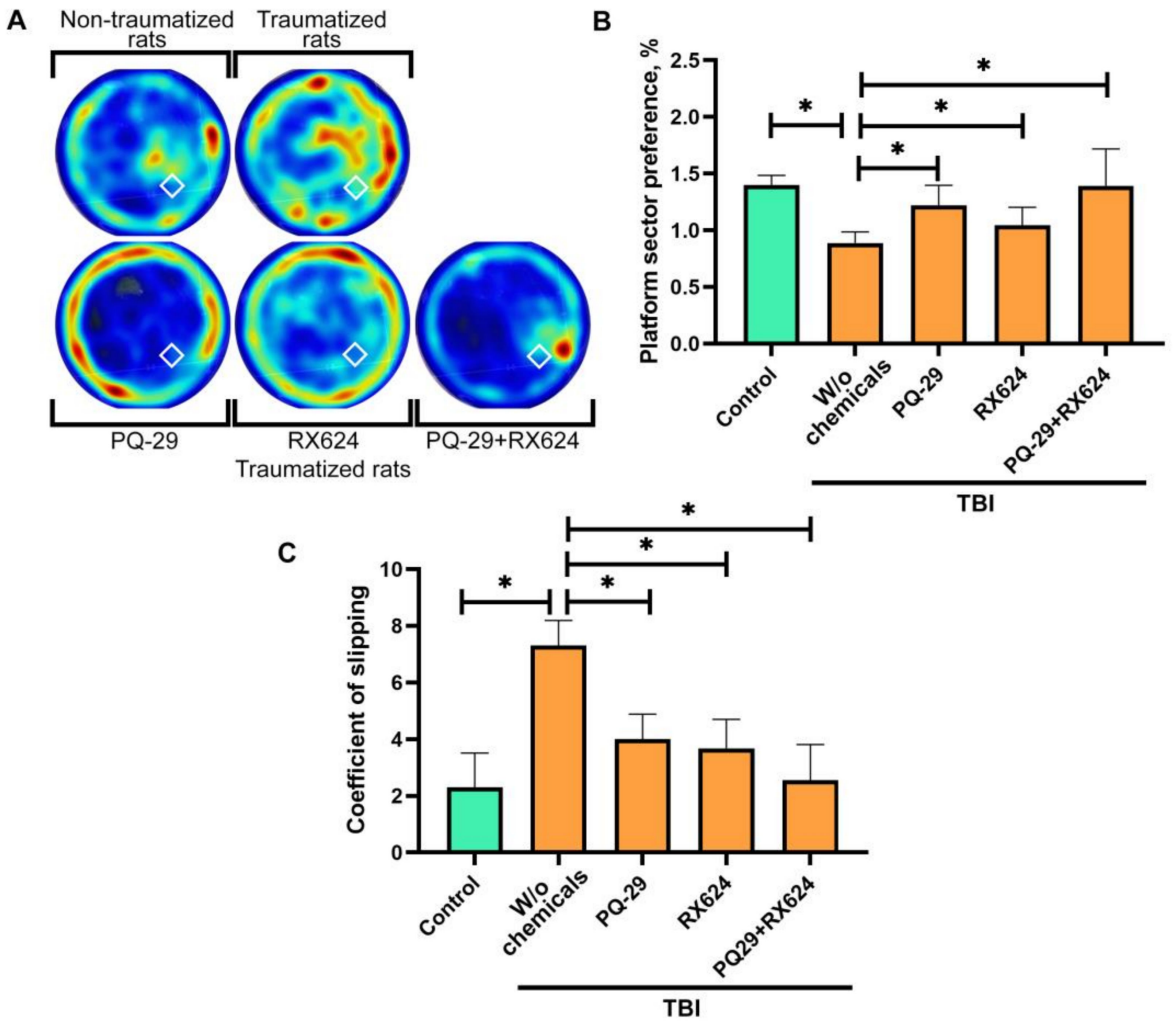
The results of the analysis of memory and motor function in traumatized rats after therapy with RX624 and PQ-29. The rats were divided into 5 groups: the control group (control), which contained naive animals, and 4 TBI groups containing traumatized animals that received intraperitoneal injections of drugs in accordance with the captions in the figure. (**A**) Memory function analysis was performed using the Morris water test. Heat maps of the movements of rats during the search for a platform are presented. The location of the escape platform during training is marked with a white square (**B**) The result of the analysis of the animals’ strategy used to find the platform in the Morris test is presented. The bar chart illustrates the ratio of the time spent by animals in the target sector to the average time spent in other sectors. (**C**) The state of the motor function of the animals was analyzed using the beam-walking test. Based on the results obtained, the slip coefficient was determined; the results are presented in the form of a bar chart. The mean values ± standard deviations are presented; differences are significant at * *p* < 0.05 (Mann—Whitney test).

To analyze memory function, we used the Morris water test (Figure 4A,B). We found that the treatment of traumatized animals using the combination of RX624 and PQ-29 resulted in the rats adopting a more effective platform searching strategy, and the rats remained in the sector in which the platform was located for a longer period of time (Figure 4A). The ratio of time spent in the target sector by the traumatized rats after receiving combined therapy to the average time spent in other sectors was 1.39 ± 0.29 (Figure 4B), which was almost identical to the corresponding parameter for non-traumatized animals (1.40 ± 0.09) and was almost twofold higher than that for traumatized animals that were not subjected to the treatment (0.88 ± 0.11). Additionally, we noted that the ratio of the duration of stay in the sector with the platform to the duration of stays in other sectors for traumatized rats that underwent monotherapy was 1.22 ± 0.16 (after PQ-29 therapy) and 1.04 ± 0.14 (after RX624 therapy) (Figure 4B).

In addition to the memory function, we evaluated the motor function of the animals. To this end, we used the beam-walking test and found that the treatment of the animals with the combination of RX624 and PQ-29 effectively prevented the development of motor deficits in traumatized animals. Thus, the coefficient of slipping of traumatized rats that did not undergo a course of treatment was 7.3 ± 0.9, and the coefficient of slipping was 2.55 ± 1.26 in animals after a course of combined therapy, which is comparable to the coefficient of slipping recorded for the group of untraumatized animals: 2.3 ± 1.21 (Figure 4B). For traumatized animals treated with monotherapy, this parameter was 4 ± 1 (in the group treated with PQ-29 injections) and 3.68 ± 1.02 (in the group treated with RX624).

**Figure 5:**
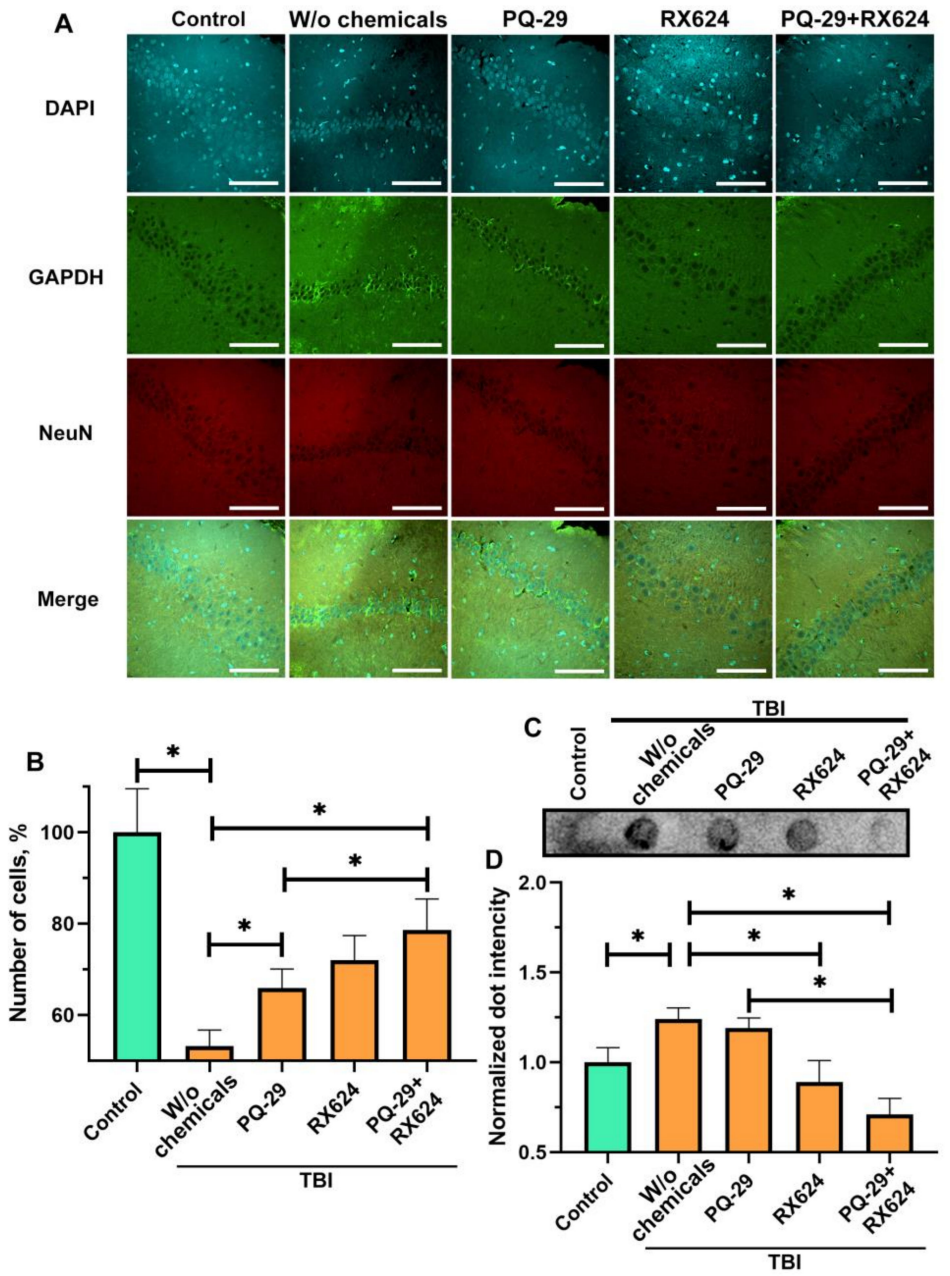
Analysis of the number of GAPDH-containing aggregates in the hippocampus of traumatized rats after a therapeutic course with the use of RX624 and PQ-29. The rats were divided into 5 groups: the control group (control), consisting of naïve animals, and 4 TBI groups, consisting of traumatized animals that received intraperitoneal injections of drugs in accordance with the captions in the figure. (**A**) The confocal microscopy data are presented. Rats’ brains from different groups were sliced and stained with DAPI (cyan) and antibodies against GAPDH (green) and NeuN (red). The CA1 hippocampus field is shown. Scale bar 10 μm. (**B**) The number of cell nuclei stained with DAPI was normalized to that in the hippocampus of control animals and presented as histogram bars. For each group, no fewer than 500 cells were counted. (**C**) Result of GAPDH aggregate quantity analysis in the rat hippocampus by filter trapping, followed by hybridization with specific anti-GAPDH antibodies. Representative image shown. (**D**) The result of the quantification of dot intensity obtained from the analysis of the amount of GAPDH. The results were normalized to the mean GAPDH staining intensity in the hippocampi of naive rats. The mean values ± standard deviations of the results of the analysis of at least three rats from each group are shown; differences are significant at * *p* < 0.05 (Mann–Whitney test).

Because, previously, we found that RX624 and PQ-29 interfered with the formation of GAPDH-containing aggregates, we sought to test whether combination therapy using both drugs would be effective in blocking GAPDH aggregation in the brains of traumatized animals. For this purpose, we prepared histological preparations for the CA1 field of the hippocampus and stained them with antibodies against NeuN (to verify the neuronal phenotype of the visualized cells), antibodies against GAPDH, and also with DAPI for nuclear imaging (Figure 5A). The images obtained using a confocal fluorescent microscope show that the maximum number of GAPDH-containing aggregate-like accumulations was observed in the brain tissue of traumatized rats that did not receive treatment. At the same time, all therapies led to a decrease in the number of GAPDH-containing aggregates, and the most effective way to prevent the formation of such complexes was the use of PQ-29, as well as the combined use of PQ-29 and RX624 (Figure 5A). We also counted the number of neurons in the CA1 field of the hippocampus and found that all therapies prevented the loss of neurons in injured animals. At the same time, therapy with RX624, as well as combination therapy with PQ-29 and RX624, turned out to be the most effective. In these cases, 72% and 78.6% of neurons, respectively, were saved (versus 53.2% of neurons being preserved in injured, untreated animals, Figure 5B).

Using the filter trap assay, we analyzed the number of GAPDH aggregates in the hippocampus of traumatized animals that underwent various therapeutic courses. It was found that the number of GAPDH-containing aggregates in the hippocampus of traumatized rats after receiving combination therapy was reduced by more than 40% compared to traumatized rats receiving no therapy (Figure 5C,D). Of note, monotherapy was not as effective—the reductions in the number of GAPDH-containing aggregates were 5% with PQ-29 therapy and 28% with RX624.

Thus, we found a trend towards an improvement in the behavioral parameters of traumatized animals administered combination therapy compared to those administered monotherapy.

## 4. Discussion

The mechanisms of secondary damage following TBI represent an extensive network of molecular interactions, many of which remain unexplored [8]. Nevertheless, it is obvious that the most promising mechanisms are therapeutic strategies that imply a combined effect on various targets. Such targets may include excitotoxicity [29] and calcium homeostasis [30], oxidative stress and mitochondrial dysfunction [31,32], neuroinflammation [33], and many others. Usually, anti-inflammatory, analgesic, and nootropic drugs are used in the clinic for rehabilitation following TBI. Neuroprotective compounds are used less often; however, many researchers agree that their use is necessary for effective treatment [34]. In the framework of the present study, we chose chemical compounds that target two druggable compounds: GAPDH and Hsp70. These proteins are involved in the regulation of apoptosis [35,36], protein homeostasis [19], response to oxidative stress [37], and the formation of toxic aggregates [38,39].

We previously demonstrated the efficacy of RX624 as an inhibitor of GAPDH aggregation in an in vitro model of oxidative stress [22] and in an in vitro TBI model [23]. It should be noted that this approach to the treatment of neurodegenerative processes has become increasingly popular in recent years. Thus, compounds that prevent the participation of GAPDH in aggregation processes in Huntington’s disease, such as deprenyl [20], and in oxidative stress accompanying Alzheimer’s disease, such as GAI [40], have been proposed as neuroprotective agents. A comparable therapeutic approach has been proposed for the treatment of dementia. Furthermore, the compounds ONO-1603 and tacrine inhibited the expression of the GAPDH gene and prevented the development of symptoms characteristic of age-related dementia [41,42]. It is likely that the effectiveness of these drugs, at least partially, was due to the fact that a decrease in the expression of the GAPDH gene also led to a reduction in the number of GAPDH-containing aggregates. We believe that the inhibition of GAPDH aggregation can be a universal therapeutic technique that is effective against neurodegenerative processes of various origins.

Another compound that we employed was an Hsp70 synthesis inducer, PQ-29. This compound was previously tested on models of TBI [15] and Alzheimer’s disease [16]. The chemical stimulation of the synthesis of chaperone proteins to increase the viability of neurons has already been tested in various models of neurodegenerative diseases. Thus, the drug geranyl–geranyl–acetone demonstrated efficacy in an animal model of TBI [43]. Moreover, the well-known inducer of chaperone synthesis arimoclomol is already used in clinics for the treatment of Niemann–Pick disease [44]. According to this point of view, the use of chaperone synthesis inducers as neuroprotectors seems to be a promising approach.

Multitarget therapy for TBI is gaining increasing attention, and hundreds of new approaches have been proposed over past 10 years [45], but among the published studies, only a third show an increase in the effectiveness of the combined use of drugs compared to monotherapy. One of the key criteria for potentially successful multitarget therapy is that each component of the therapy must have mono-agentic activity without cross-resistance [46]. Cross-resistance may occur if therapeutic compounds used together act in a similar manner. The therapy we proposed using PQ-29 and RX624 met the above criterion, as the targets of therapy in this case were intracellular heat shock proteins and extracellular GAPDH, and the mechanisms of the effects exerted by the compounds were completely different.

Nevertheless, some limitations of this study should be noted. First, this study concerns the use of a model based on rat glioma cells. We assumed that the effects of therapy may be different on differentiated neurons. In addition, one must be very careful about GAPDH inhibition. It is the basic enzyme of the glycolytic cycle, and its inhibition can lead to multiple consequences. That is why, in our early studies, we made sure that the PX624 compound did not affect the glycolytic activity of the enzyme [47]. Another limitation of the applicability of the results obtained relates to the small number of animals participating in the experiments. Undoubtedly, in the process of optimizing the described combination therapy approach, more specific behavioral tests are needed. Finally it is still difficult to state how exactly the used TBI model in rats corresponds to a specific clinical picture for patients. Most likely, similar therapeutic approaches can be applied for severe TBI according to characteristics of the weight drop TBI model [25]. It is very important that, in the present study, both compounds had an effect on both the restoration of cell proliferation (Figure 2) and the development of apoptosis (Figure 3) in an in vitro model. Similarly, in an in vivo model, both compounds prevented the development of memory and motor impairments. Similar results indicate the influence of secondary damage on various mechanisms of development. At the same time, in all the experiments we performed, the use of a combined approach tended to be more effective than monotherapy.

## 5. Conclusions

The combined use of a protein aggregation inhibitor and an inducer of chaperone synthesis can be an effective approach to preventing the secondary death of brain tissue following TBI. In some of the experiments performed, combination therapy using a similar combination of drugs (RX624 and PQ-29) tended to be more effective than monotherapy with PQ-29. Nevertheless, to transfer such a form of therapy to clinics, it is necessary to study the time frame of the therapeutic window, and it is also essential to test both the compounds and their combination in preclinical trials.

## Data Availability

The data are available within the article.
